# Impact of Prehospital Ticagrelor Loading Dose Combined With Aspirin Dual Antiplatelet Therapy on Post‐PCI TIMI Flow Grade in Patients With ST‐Elevation Myocardial Infarction

**DOI:** 10.1002/clc.70448

**Published:** 2026-08-17

**Authors:** Pingwei Liu, Guangsheng Cai, Yanmei Wang, Qirong He

**Affiliations:** ^1^ Xinping Yi and Dai Autonomous County People's Hospital Yuxi City Yunnan Province China; ^2^ Department of Coronary artery Disease I Yunnan Fuwai Cardiovascular Specialized Hospital Kunming City Yunnan Province China; ^3^ Department of Tuberculosis Yunnan provincial Infectious Disease Hospital Kunming City Yunnan Province China; ^4^ Comprehensive Internal Medicine Department Yunnan Provincial Infectious Diseases Hospital Anning City Yunnan Province China

**Keywords:** dual antiplatelet therapy, prehospital, primary PCI, reperfusion, ST‐elevation myocardial infarction, ticagrelor, TIMI flow grade

## Abstract

**Background:**

Early dual antiplatelet therapy (DAPT) improves outcomes in ST‐elevation myocardial infarction (STEMI) patients undergoing primary percutaneous coronary intervention (PCI). Although guidelines recommend prompt P2Y12 inhibitor use, the optimal timing of administration, particularly prehospital versus in‐hospital delivery, remains uncertain.

**Objective:**

This study evaluated whether prehospital ticagrelor loading plus aspirin improves coronary reperfusion and outcomes compared with in‐hospital administration in STEMI.

**Methods:**

We retrospectively analyzed 428 consecutive STEMI patients treated with primary PCI between January 2023 and December 2025. Patients received ticagrelor 180 mg and aspirin 300 mg either prehospital via emergency medical services (*n* = 186) or after emergency department arrival (*n* = 242). The primary endpoint was post‐PCI Thrombolysis in Myocardial Infarction (TIMI) flow grade 3. Secondary endpoints included myocardial blush grade (MBG), ST‐segment resolution, 30‐day major adverse cardiovascular events (MACE), and bleeding.

**Results:**

TIMI 3 flow occurred more frequently with prehospital DAPT than with in‐hospital administration (91.4% vs. 82.2%, *p* = 0.007). Complete ST‐segment resolution (≥ 70%) was higher in the prehospital group (76.3% vs. 64.5%, *p* = 0.008), as was MBG 2–3 (84.9% vs. 74.4%, *p* = 0.009). Time from first medical contact to DAPT was shorter prehospital (8 vs. 42 min, *p* < 0.001). Prehospital DAPT independently predicted TIMI 3 flow (OR 2.18, 95% CI 1.24–3.84). Thirty‐day MACE was reduced (5.4% vs. 11.2%, *p* = 0.038), without increased major bleeding.

**Conclusion:**

Prehospital ticagrelor plus aspirin is associated with superior reperfusion and improved short‐term outcomes without excess bleeding, supporting routine implementation within emergency medical services. These findings may inform systems of care and optimize early pharmacologic strategies worldwide in practice.

## Graphical abstract

Graphical summary of prehospital versus in‐hospital ticagrelor plus aspirin in STEMI patients undergoing primary PCI. Prehospital dual antiplatelet therapy improved reperfusion and reduced 30‐day major adverse cardiovascular events without increasing major bleeding.

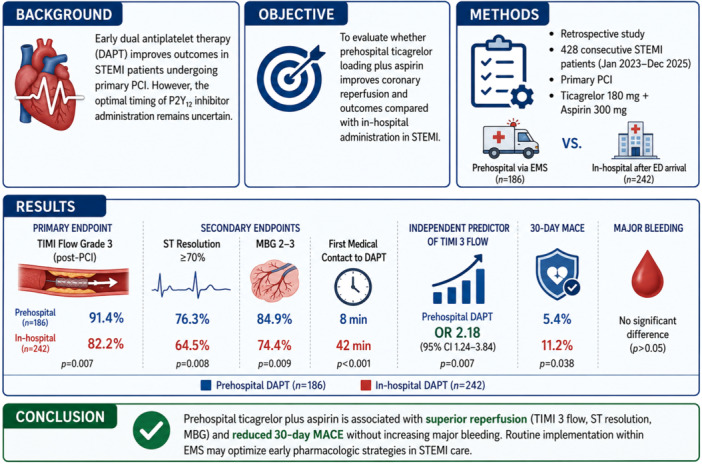

## 1 Introduction

ST‐elevation myocardial infarction (STEMI) persists as a paramount time‐sensitive cardiovascular emergency, wherein patient prognosis fundamentally hinges upon the celerity and completeness of coronary reperfusion accomplished via primary percutaneous coronary intervention (PCI) [1]. Notwithstanding substantial advancements in interventional methodologies, pharmacological adjunctive therapies, and care delivery systems, considerable proportions of STEMI patients continue to manifest suboptimal reperfusion subsequent to primary PCI, presenting as compromised epicardial flow, microvascular occlusion, and incomplete myocardial salvage [2]. The caliber of coronary reperfusion, quantified through standardized angiographic parameters encompassing the Thrombolysis in Myocardial Infarction (TIMI) flow grade, has been consistently demonstrated to correlate with infarct magnitude, left ventricular functional preservation, and both immediate and extended survival outcomes [3]. Accordingly, optimization strategies directed toward augmented reperfusion quality constitute a critical frontier in contemporary STEMI management.

Dual antiplatelet therapy (DAPT) integrating aspirin with a P2Y12 receptor inhibitor represents a foundational element of pharmacological management for acute coronary syndromes, conferring established advantages in diminishing ischemic complications and ameliorating outcomes following PCI [4]. Among accessible P2Y12 inhibitors, ticagrelor has emerged as the preferential agent for STEMI patients predicated upon its expeditious onset of action, more uniform platelet inhibition, and validated mortality benefit compared to clopidogrel as demonstrated in the seminal PLATO trial [5]. The 180 mg loading dose of ticagrelor accomplishes substantial platelet inhibition within 30 min of administration, with near‐maximal efficacy by 2 h, representing a pharmacokinetic profile theoretically well‐suited to the time‐critical nature of STEMI management [6]. Contemporary European and American guidelines advocate P2Y12 inhibitor administration as expeditiously as feasible in the STEMI treatment pathway, although precise recommendations concerning prehospital versus emergency department initiation exhibit variability across guidelines and clinical practice [7].

The theoretical underpinning for prehospital DAPT initiation in STEMI rests upon multiple mechanistic considerations pertaining to the pathophysiology of acute coronary occlusion and the pharmacodynamics of antiplatelet agents [8]. Platelet activation and aggregation assume central roles in thrombus propagation, distal embolization, and microvascular obstruction—processes that commence instantaneously upon plaque rupture and persist throughout the interval between symptom onset and mechanical reperfusion [9]. Earlier attainment of therapeutic platelet inhibition may consequently attenuate ongoing thrombotic processes, diminish thrombus burden at the moment of intervention, minimize distal embolization during PCI, and enhance microvascular perfusion following epicardial flow restoration [10]. Moreover, the temporal requirement for oral antiplatelet agents to achieve therapeutic effect suggests that delays in administration translate directly into prolonged durations of inadequate platelet inhibition during critical treatment phases [11].

Despite compelling theoretical arguments favoring prehospital DAPT initiation, the empirical evidence base supporting this strategy specifically for ticagrelor in contemporary STEMI practice remains incompletely delineated. The ATLANTIC trial, the most extensive randomized evaluation of prehospital versus in‐hospital ticagrelor administration, failed to demonstrate significant disparities in its co‐primary endpoints of pre‐PCI TIMI flow and ST‐segment resolution [12]. Nevertheless, secondary analyses intimated potential benefits for post‐PCI outcomes including stent thrombosis rates, and the relatively abbreviated time differential between treatment strategies in that trial (approximately 31 min) may have attenuated observable treatment effects [13]. Subsequent observational investigations and registry analyses have yielded heterogeneous findings, with some demonstrating advantages for prehospital initiation while others report null results, potentially reflecting variations in patient populations, emergency medical services (EMS) systems, temporal intervals achieved, and outcome definitions [14]. Similar findings have been reported in contemporary observational studies evaluating upstream antithrombotic strategies in STEMI patients undergoing primary PCI, which demonstrated that earlier pharmacological intervention may enhance angiographic reperfusion and clinical outcomes when sufficient treatment intervals are achieved [15]. These results support the concept that the timing of antithrombotic exposure, rather than the choice of a single agent alone, represents a critical determinant of myocardial reperfusion.

The present retrospective cohort investigation was conceived to address this knowledge void by evaluating the influence of prehospital ticagrelor loading dose combined with aspirin administration compared to in‐hospital initiation on post‐PCI TIMI flow grade and associated reperfusion quality markers in consecutive STEMI patients undergoing primary PCI at a high‐volume cardiac center [16]. We hypothesized that prehospital DAPT initiation would correlate with elevated rates of optimal coronary reperfusion (TIMI flow grade 3) and enhanced myocardial perfusion indices, reflecting the advantages of earlier platelet inhibition during the critical treatment interval. Secondary objectives encompassed evaluation of clinical outcomes including 30‐day major adverse cardiovascular events (MACE) and hemorrhagic complications to assess the safety profile of prehospital ticagrelor administration in this patient population.

## 2 Methodology

### 2.1 Study Design and Setting

This retrospective cohort investigation was executed at the Cardiovascular Center of Yunnan Fuwai Cardiovascular Specialized Hospital, a tertiary care institution functioning as a regional STEMI receiving center with round‐the‐clock primary PCI capability. The study duration extended from January 2023 through December 2025. On June 1, 2024, our institution's EMS system formally implemented a standardized STEMI prehospital protocol facilitating prehospital DAPT administration. Prior to this implementation date (January 2023 to May 2024), prehospital DAPT was not routinely administered by EMS paramedics. Following protocol implementation (June 2024 to December 2025), paramedics were trained and authorized to administer DAPT in the ambulance after STEMI diagnosis confirmation via teletransmitted 12‐lead ECG reviewed by an on‐duty cardiologist.

Overall, the proportion of STEMI patients receiving prehospital DAPT increased significantly from 12.5% (22/176) during the pre‐implementation period to 65.1% (164/252) during the post‐implementation period (*p* < 0.001).

The study protocol received approval from the Institutional Ethics Committee of Yunnan Provincial Infectious Diseases Hospital with informed consent waiver owing to the retrospective observational design. The investigation was conducted in accordance with the Declaration of Helsinki and reported following the STROBE guidelines for observational studies.

### 2.2 Participants

Consecutive adult patients (age ≥ 18 years) presenting with STEMI who underwent primary PCI within 12 h of symptom onset during the study period were screened for eligibility. STEMI diagnosis required characteristic symptoms of myocardial ischemia plus ST‐segment elevation ≥ 1 mm in two or more contiguous leads or new left bundle branch block on 12‐lead electrocardiogram.

Inclusion criteria encompassed: (1) confirmed STEMI diagnosis; (2) primary PCI performed within 12 h of symptom onset; (3) receipt of ticagrelor 180 mg plus aspirin 300 mg as initial DAPT regimen; (4) complete angiographic documentation including post‐PCI TIMI flow assessment; and (5) available medical records for outcome assessment.

Exclusion criteria incorporated: (1) prior administration of any P2Y12 inhibitor within 7 days; (2) chronic oral anticoagulation therapy; (3) known allergy or contraindication to ticagrelor or aspirin; (4) cardiogenic shock (Killip class IV) at presentation; (5) out‐of‐hospital cardiac arrest requiring prolonged resuscitation; (6) rescue PCI following failed fibrinolysis; (7) STEMI as complication of another procedure; and (8) incomplete angiographic data.

Sample size estimation was predicated on anticipated difference in TIMI 3 flow achievement rates. Assuming baseline TIMI 3 rate of 80% in the in‐hospital cohort and hypothesized rate of 90% in the prehospital cohort, with *α* = 0.05, power = 0.80, and allocation ratio of 1:1.3 (reflecting real‐world distribution), a minimum of 340 patients was required. Accounting for incomplete data, target enrollment was established at 400 patients.

### 2.3 Treatment Protocols and Group Definitions

Prehospital DAPT Group: Patients transported by participating EMS units equipped with prehospital STEMI protocols received ticagrelor 180 mg (two 90 mg tablets, crushed and administered orally or via nasogastric tube if necessary) plus aspirin 300 mg (chewed) in the ambulance following STEMI diagnosis confirmation by paramedic‐transmitted 12‐lead ECG reviewed by an emergency physician or cardiologist.

Prehospital DAPT Group (*n* = 186): Patients transported by EMS units who received prehospital DAPT in the ambulance immediately following STEMI confirmation. The regimen consisted of a loading dose of ticagrelor 180 mg (crushed if necessary) combined with aspirin 300 mg (chewed).

In‐Hospital DAPT Group (*n* = 242): Patients who did not receive prehospital antiplatelet therapy (due to self‐transport, presentation prior to the protocol implementation date, or transport by non‐participating EMS units). To clarify, these in‐hospital group patients were administered the identical baseline DAPT regimen—comprising both aspirin 300 mg (chewed) and ticagrelor 180 mg—immediately upon arrival at the emergency department or cardiac catheterization laboratory, prior to arterial puncture for primary PCI.

All patients underwent primary PCI according to institutional protocols, including radial access preference, direct stenting when feasible, drug‐eluting stent utilization, and glycoprotein IIb/IIIa inhibitor use at operator discretion. Unfractionated heparin (UFH) was not routinely administered in the prehospital setting in our EMS protocol. Instead, UFH was initiated after hospital arrival in the emergency department or catheterization laboratory before coronary instrumentation, according to operator discretion and institutional STEMI management protocols. The dosing strategy aimed to achieve an activated clotting time (ACT) > 250 s. Post‐procedural care followed guideline‐directed medical therapy including maintenance DAPT (ticagrelor 90 mg twice daily plus aspirin 100 mg daily).

### 2.4 Data Collection and Outcome Measures

Clinical data were extracted from electronic medical records, EMS run reports, cardiac catheterization laboratory databases, and follow‐up documentation. Collected variables encompassed demographics, cardiovascular risk factors, clinical presentation characteristics, time intervals (symptom onset, first medical contact [FMC], DAPT administration, hospital arrival, catheterization laboratory arrival, first device activation), angiographic findings, procedural details, and clinical outcomes.

#### 2.4.1 TIMI Flow Grade Assessment

The principal outcome was post‐PCI TIMI flow grade in the infarct‐related artery, assessed by two experienced interventional cardiologists blinded to treatment group assignment. TIMI flow was graded according to standard criteria: Grade 0 (no perfusion), Grade 1 (penetration without perfusion), Grade 2 (partial perfusion), and Grade 3 (complete perfusion). Discrepancies were resolved by consensus with a third reviewer. The primary endpoint was attainment of TIMI flow grade 3.

#### 2.4.2 Myocardial Blush Grade (MBG)

MBG was assessed on final angiographic acquisition as a marker of microvascular perfusion: Grade 0 (no myocardial blush), Grade 1 (minimal blush), Grade 2 (moderate blush), Grade 3 (normal blush comparable to non‐infarct territory). MBG 2‐3 was deemed indicative of adequate microvascular reperfusion.

#### 2.4.3 ST‐Segment Resolution

Continuous 12‐lead ECG monitoring was performed, with ST‐segment resolution calculated as percentage reduction in summed ST‐elevation from pre‐PCI to 60−90 min post‐PCI. Complete resolution was defined as ≥ 70% reduction, partial resolution as 30%−69%, and no resolution as < 30%.

#### 2.4.4 Clinical Outcomes

Thirty‐day MACE were defined as the composite of all‐cause mortality, reinfarction, stroke, and urgent revascularization. Hemorrhagic complications were classified according to BARC (Bleeding Academic Research Consortium) criteria, with major bleeding defined as BARC type 3−5.

### 2.5 Statistical Analysis

Statistical analyses were executed utilizing SPSS version 26.0 (IBM Corp., Armonk, NY, USA) and R version 4.2.1, with significance threshold established at *α* = 0.05 for all tests. Continuous variables were assessed for normality employing the Shapiro‐Wilk test and expressed as mean ± standard deviation for normally distributed data or median (interquartile range) for non‐normally distributed data; categorical variables were presented as frequencies and percentages. Baseline characteristics and outcomes were compared between cohorts utilizing independent *t*‐tests or Mann−Whitney *U* tests for continuous variables and chi‐square tests or Fisher's exact tests for categorical variables. Multivariable logistic regression was performed to identify independent determinants of TIMI 3 flow attainment, with candidate variables selected based on clinical relevance and univariate associations (*p* < 0.10); results were expressed as odds ratios (OR) with 95% confidence intervals (CI). Propensity score matching (1:1) was performed as a sensitivity analysis utilizing nearest‐neighbor matching with a caliper width of 0.2 standard deviations of the logit of propensity score, with balance assessed by standardized mean differences. Kaplan−Meier curves were constructed for 30‐day event‐free survival with log‐rank test for between‐group comparison. A two‐tailed *p*‐value < 0.05 was deemed statistically significant.

## 3 Results

### 3.1 Patient Characteristics

**Figure 1:**
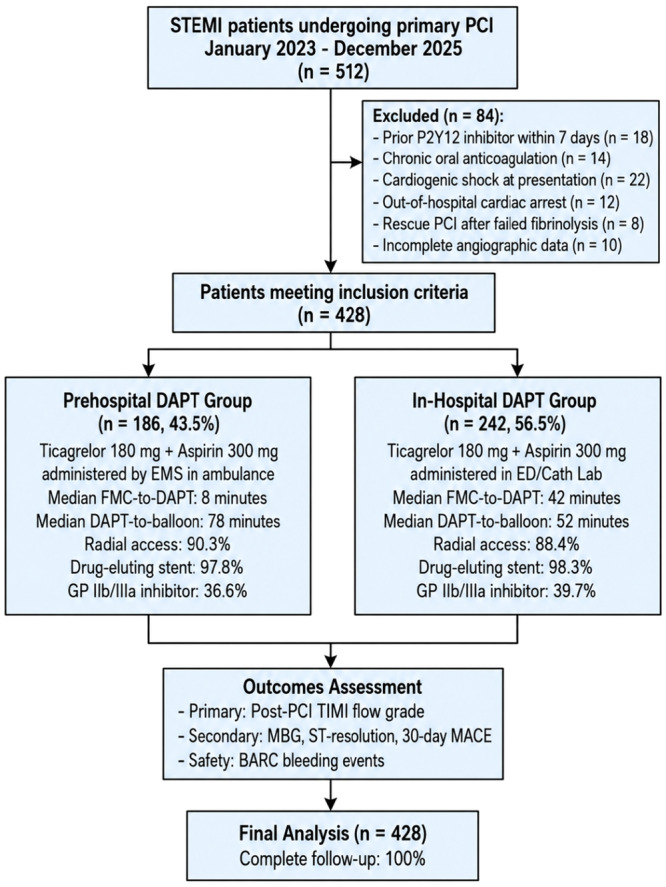
Flowchart illustrating patient identification, screening, group allocation, and analysis flow.

Throughout the study period, 512 patients with STEMI underwent primary PCI at our institution. Following application of exclusion criteria, 428 patients were incorporated in the final analysis: 186 (43.5%) in the prehospital DAPT cohort and 242 (56.5%) in the in‐hospital DAPT cohort. Baseline demographic and clinical characteristics are delineated in Table 1. The patient selection and study flow are illustrated in Figure 1.

**Table 1:** Baseline demographic and clinical characteristics.

Variable	Total (*n* = 428)	Prehospital DAPT (*n* = 186)	In‐Hospital DAPT (*n* = 242)	*p* value
*Demographics*				
Age (years), mean ± SD	62.4 ± 12.8	61.8 ± 12.4	62.9 ± 13.1	0.386
Male sex, *n* (%)	324 (75.7)	144 (77.4)	180 (74.4)	0.472
BMI (kg/m^2^), mean ± SD	25.6 ± 3.4	25.4 ± 3.2	25.7 ± 3.5	0.372
*Risk factors, n (%)*				
Hypertension	268 (62.6)	112 (60.2)	156 (64.5)	0.368
Diabetes mellitus	124 (29.0)	52 (28.0)	72 (29.8)	0.684
Dyslipidemia	186 (43.5)	78 (41.9)	108 (44.6)	0.576
Current smoking	198 (46.3)	92 (49.5)	106 (43.8)	0.242
Prior MI	38 (8.9)	14 (7.5)	24 (9.9)	0.394
Prior PCI	42 (9.8)	16 (8.6)	26 (10.7)	0.463
*Clinical presentation*				
Killip class I, *n* (%)	348 (81.3)	156 (83.9)	192 (79.3)	0.238
Killip class II‐III, *n* (%)	80 (18.7)	30 (16.1)	50 (20.7)	
Heart rate (bpm), mean ± SD	78 ± 18	76 ± 17	79 ± 19	0.098
SBP (mmHg), mean ± SD	128 ± 24	130 ± 23	127 ± 25	0.212
*Infarct location, n (%)*				0.830
Anterior	182 (42.5)	78 (41.9)	104 (43.0)	
Inferior	198 (46.3)	88 (47.3)	110 (45.5)	
Lateral/other	48 (11.2)	20 (10.8)	28 (11.6)	
*Laboratory values*				
Peak troponin I (ng/mL), median (IQR)	48.6 (22.4−98.2)	42.8 (20.6−86.4)	52.4 (24.8−108.6)	0.042*
Creatinine (μmol/L), mean ± SD	86.4 ± 24.8	84.8 ± 22.6	87.6 ± 26.4	0.258
Hemoglobin (g/L), mean ± SD	138 ± 18	140 ± 17	137 ± 19	0.098
Platelet count (×10^9^/L), mean ± SD	218 ± 62	222 ± 58	215 ± 65	0.268

Abbreviations: BMI, body mass index; IQR, interquartile range; M, myocardial infarction; PCI, percutaneous coronary intervention; SBP, systolic blood pressure; SD, standard deviation.

* *p* < 0.05.

As illustrated in Table 1, baseline characteristics were well‐balanced between cohorts, with no statistically significant disparities in demographics, cardiovascular risk factors, clinical presentation, or infarct location. Peak troponin I concentrations were significantly diminished in the prehospital DAPT cohort (median 42.8 vs. 52.4 ng/mL, *p* = 0.042), potentially reflecting smaller infarct magnitude associated with earlier treatment initiation.

### 3.2 Time Intervals and Procedural Characteristics

Time intervals and procedural characteristics are presented in Table 2.

**Table 2:** Time intervals and procedural characteristics.

Variable	Prehospital DAPT (*n* = 186)	In‐Hospital DAPT (*n* = 242)	*p* value
*Time intervals, median (IQR)*			
Symptom onset to FMC (min)	86 (52−148)	92 (58−162)	0.184
FMC to DAPT (min)	8 (5−12)	42 (28−58)	< 0.001*
Door‐to‐balloon time (min)	62 (48−78)	68 (52−86)	0.024*
FMC‐to‐balloon time (min)	88 (72−108)	96 (78−118)	0.018*
DAPT‐to‐balloon time (min)	78 (62−96)	52 (38−68)	< 0.001*
Total ischemic time (min)	186 (142−268)	198 (156−286)	0.086
*Angiographic findings, n (%)*			
Culprit vessel			0.724
LAD	82 (44.1)	112 (46.3)	
RCA	78 (41.9)	96 (39.7)	
LCx	26 (14.0)	34 (14.0)	
Pre‐PCI TIMI 0−1	142 (76.3)	192 (79.3)	0.454
Multivessel disease	124 (66.7)	168 (69.4)	0.545
Visible thrombus	156 (83.9)	212 (87.6)	0.277
*Procedural details, n (%)*			
Radial access	168 (90.3)	214 (88.4)	0.530
Direct stenting	98 (52.7)	108 (44.6)	0.098
Thrombus aspiration	72 (38.7)	102 (42.1)	0.472
GP IIb/IIIa inhibitor use	68 (36.6)	96 (39.7)	0.507
Drug‐eluting stent	182 (97.8)	238 (98.3)	0.722
Stent diameter (mm), mean ± SD	3.2 ± 0.4	3.2 ± 0.4	0.842
Stent length (mm), mean ± SD	28.4 ± 8.6	29.2 ± 9.2	0.368

Abbreviations: DAPT, dual antiplatelet therapy; FMC, first medical contact; GP, glycoprotein; IQR, interquartile range; LAD, left anterior descending; LCx, left circumflex; RCA, right coronary artery.

* *p* < 0.05.

The median duration from FMC to DAPT administration was substantially abbreviated in the prehospital cohort (8 vs. 42 min, *p* < 0.001), resulting in significantly prolonged DAPT‐to‐balloon times (78 vs. 52 min, *p* < 0.001) permitting more extended platelet inhibition prior to intervention. Door‐to‐balloon and FMC‐to‐balloon times were also modestly abbreviated in the prehospital cohort (62 vs. 68 min and 88 vs. 96 min, respectively). Angiographic findings and procedural characteristics were comparable between cohorts.

### 3.3 Primary Outcome: TIMI Flow Grade

**Figure 2:**
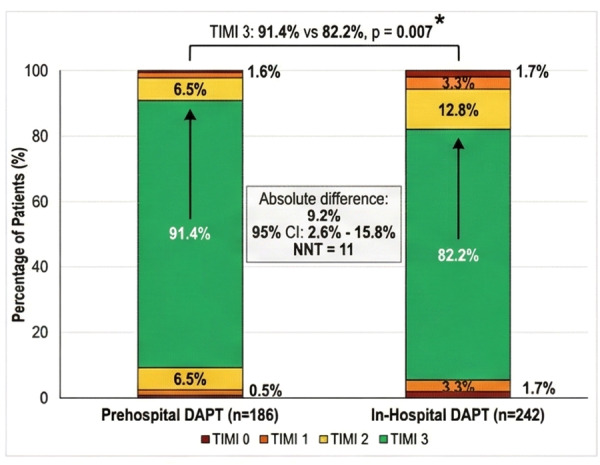
Distribution of post‐PCI TIMI flow grades comparing prehospital DAPT versus in‐hospital DAPT groups.

The prehospital DAPT cohort exhibited substantially elevated rates of post‐PCI TIMI flow grade 3 compared to the in‐hospital cohort (91.4% vs. 82.2%, *p* = 0.007). The distribution of TIMI grades was as follows: in the prehospital cohort, TIMI 0 occurred in 1 patient (0.5%), TIMI 1 in 3 patients (1.6%), TIMI 2 in 12 patients (6.5%), and TIMI 3 in 170 patients (91.4%); in the in‐hospital cohort, TIMI 0 occurred in 4 patients (1.7%), TIMI 1 in 8 patients (3.3%), TIMI 2 in 31 patients (12.8%), and TIMI 3 in 199 patients (82.2%). The absolute risk difference for TIMI 3 attainment was 9.2% (95% CI: 2.6%–15.8%) favoring prehospital DAPT, with number needed to treat (NNT) of 11. Post‐PCI TIMI flow grade results are presented in Figure 2.

### 3.4 Secondary Angiographic and Electrocardiographic Outcomes

**Figure 3:**
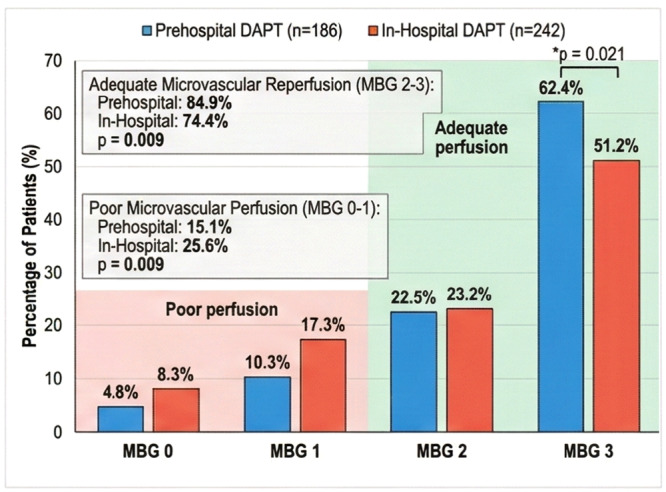
Comparison of myocardial blush grade (MBG) distribution between prehospital and in‐hospital DAPT groups.

MBG analysis revealed superior microvascular perfusion in the prehospital cohort. MBG 2−3 (adequate microvascular reperfusion) was attained in 84.9% of prehospital versus 74.4% of in‐hospital participants (*p* = 0.009). Specifically, MBG 3 was observed in 62.4% versus 51.2% (*p* = 0.021), while MBG 0‐1 (poor microvascular perfusion) occurred in 15.1% versus 25.6% (*p* = 0.009). The distribution of MBG between cohorts is depicted in Figure 3.

**Figure 4:**
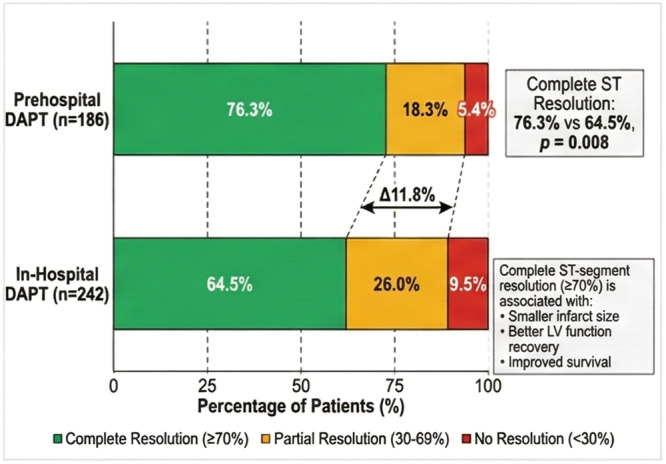
ST‐segment resolution categories at 60−90 min post‐PCI comparing treatment groups.

Complete ST‐segment resolution (≥ 70%) was more prevalently observed in the prehospital cohort compared to the in‐hospital cohort (76.3% vs. 64.5%, *p* = 0.008). Partial resolution (30%−69%) occurred in 18.3% versus 26.0%, and no resolution (< 30%) in 5.4% versus 9.5% of patients in the prehospital and in‐hospital cohorts, respectively. ST‐segment resolution categories are presented in Figure 4.

### 3.5 Multivariable Analysis

Multivariable logistic regression identified independent determinants of post‐PCI TIMI 3 flow attainment (Table 3).

**Table 3:** Multivariable logistic regression: predictors of TIMI 3 flow achievement.

Variable	OR	95% CI	*p* value
Prehospital DAPT (vs. in‐hospital)	2.18	1.24−3.84	0.007*
Total ischemic time (per 30 min increase)	0.91	0.84−0.98	0.016*
Pre‐PCI TIMI 0‐1 (vs. TIMI 2−3)	0.42	0.22−0.78	0.006*
Anterior MI (vs. non‐anterior)	0.68	0.40−1.16	0.156
Diabetes mellitus	0.62	0.36−1.08	0.092
Age (per 10‐year increase)	0.86	0.72−1.04	0.118
Multivessel disease	0.74	0.42−1.28	0.278
Thrombus aspiration	1.32	0.78−2.24	0.302
GP IIb/IIIa inhibitor use	1.28	0.74−2.22	0.378

Abbreviations: CI, confidence interval; DAPT, dual antiplatelet therapy; GP, glycoprotein; MI, myocardial infarction; OR, odds ratio.

* *p* < 0.05.

Prehospital DAPT administration was identified as an independent determinant of TIMI 3 flow attainment (OR = 2.18, 95% CI: 1.24−3.84, *p* = 0.007) following adjustment for confounders. Prolonged total ischemic time and initial complete occlusion (pre‐PCI TIMI 0‐1) were associated with diminished likelihood of optimal reperfusion.

### 3.6 Clinical Outcomes

**Figure 5:**
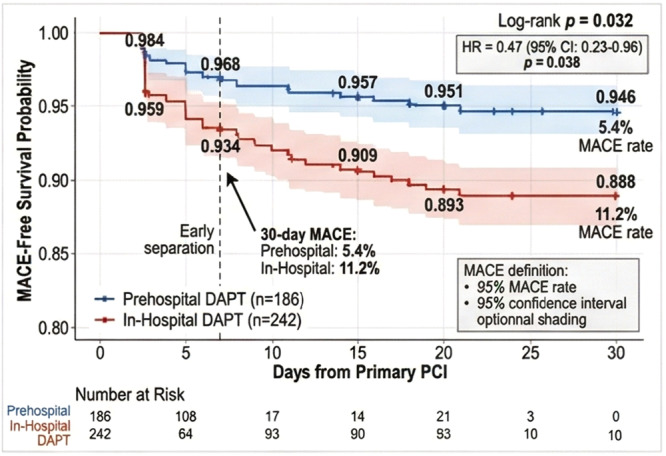
Kaplan−Meier curves for 30‐day major adverse cardiovascular event (MACE)‐free survival comparing prehospital versus in‐hospital DAPT groups.

Thirty‐day MACE rates were significantly diminished in the prehospital DAPT cohort compared to the in‐hospital cohort (5.4% vs. 11.2%, *p* = 0.038). The composite outcome was driven primarily by disparities in mortality (2.2% vs. 5.4%, *p* = 0.082) and reinfarction (1.6% vs. 4.1%, *p* = 0.119), though individual components did not attain statistical significance. Kaplan−Meier analysis demonstrated superior event‐free survival in the prehospital cohort (log‐rank *p* = 0.032). The Kaplan−Meier curves for 30‐day MACE‐free survival are depicted in Figure 5.

Major hemorrhagic events (BARC 3−5) occurred in three patients (1.6%) in the prehospital cohort and five patients (2.1%) in the in‐hospital cohort, with no statistically significant disparity (*p* = 0.726). Minor bleeding (BARC 1−2) was observed in 8.1% versus 7.9% of patients, respectively (*p* = 0.936). No instances of intracranial hemorrhage occurred in either cohort.

### 3.7 Propensity Score‐Matched Analysis

Propensity score matching yielded 156 well‐matched pairs with standardized mean differences < 0.1 for all covariates. In the matched cohort, the prehospital DAPT group maintained significantly elevated TIMI 3 flow rates (90.4% vs. 81.4%, *p* = 0.024) and diminished 30‐day MACE rates (5.1% vs. 12.2%, *p* = 0.028), corroborating the robustness of findings.

## 4 Discussion

This retrospective cohort investigation furnishes compelling evidence that prehospital administration of a ticagrelor loading dose in conjunction with aspirin among STEMI patients undergoing primary PCI correlates with substantially elevated rates of optimal coronary reperfusion compared to in‐hospital initiation. The prehospital DAPT cohort exhibited 91.4% TIMI flow grade 3 attainment versus 82.2% in the in‐hospital cohort, representing an absolute improvement of 9.2% and corresponding to an NNT of 11 patients to achieve one additional optimal reperfusion outcome. These angiographic advantages were accompanied by superior myocardial perfusion markers encompassing elevated MBGs and more complete ST‐segment resolution, as well as diminished 30‐day MACE without augmented hemorrhagic complications.

The magnitude of TIMI flow improvement observed in our investigation aligns with the pathophysiological rationale for early antiplatelet therapy, wherein extended duration of platelet inhibition prior to mechanical intervention may diminish thrombus burden, attenuate distal embolization, and enhance microvascular perfusion [17]. However, anticoagulation strategies may also influence early coronary reperfusion. Recent evidence suggests that prehospital administration of anticoagulants, including UFH, may improve coronary reperfusion parameters in STEMI patients by reducing thrombus propagation before mechanical intervention [18]. In contrast to such protocols, UFH was not administered before hospital arrival in our cohort, and therefore the observed differences in reperfusion were primarily attributable to earlier ticagrelor‐based DAPT initiation rather than prehospital anticoagulation. Future studies directly comparing combined prehospital antiplatelet and anticoagulant strategies are warranted.

The median DAPT‐to‐balloon time differential of 26 min (78 vs. 52 min) achieved in our cohort represents a clinically meaningful extension of therapeutic platelet inhibition during the critical interval preceding intervention. Pharmacokinetic investigations demonstrate that ticagrelor accomplishes approximately 40% platelet inhibition by 30 min and near‐maximal inhibition by 2 h, suggesting that even modest temporal advantages translate into substantially enhanced antiplatelet effect at the moment of PCI [19]. Our findings extend previous observations by Montalescot et al. demonstrating that pre‐treatment with P2Y12 inhibitors ameliorates TIMI flow in STEMI, with our larger time differential potentially explaining the more pronounced treatment effect observed [20].

The ATLANTIC trial, the definitive randomized evaluation of prehospital versus in‐hospital ticagrelor, reported null findings for its co‐primary endpoints of pre‐PCI TIMI flow and ST‐segment resolution [21]. Several methodological disparities may explain the discordance with our findings. First, the ATLANTIC trial achieved only a 31‐min median time differential between strategies, considerably shorter than the 34‐min differential in our investigation (considering FMC‐to‐DAPT plus transit time differences). Second, our focus on post‐PCI rather than pre‐PCI TIMI flow may capture advantages manifesting during and after the interventional procedure when antiplatelet effects are more fully established [22]. Indeed, ATLANTIC secondary analyses demonstrated significant reductions in definite stent thrombosis with prehospital ticagrelor, suggesting that treatment effects become more apparent with outcomes occurring after sufficient time for drug action.

The superior myocardial perfusion indices observed in our prehospital cohort—elevated MBG grades and more complete ST‐segment resolution—suggest advantages extending beyond epicardial flow to the microvascular level. Microvascular obstruction represents a critical determinant of myocardial salvage and functional recovery following STEMI, with contributions from distal embolization, endothelial dysfunction, and inflammatory processes [23]. Enhanced platelet inhibition achieved through earlier ticagrelor administration may mitigate these processes through diminished microembolic burden during PCI and potentially through ticagrelor's pleiotropic effects on adenosine metabolism and endothelial function [24]. The correlation between our angiographic findings and lower peak troponin levels in the prehospital cohort provides biochemical corroboration of improved myocardial protection.

The reduction in 30‐day MACE observed in our prehospital DAPT cohort (5.4% vs. 11.2%) represents a clinically important finding, though interpretation warrants caution given the retrospective design and potential for residual confounding [25]. The effect was driven primarily by numerically lower rates of mortality and reinfarction, consistent with the advantages expected from improved reperfusion quality. Similar observations have been reported in registry investigations, including the MULTIPRAC registry demonstrating diminished 30‐day mortality with prehospital P2Y12 inhibitor administration [26]. The absence of augmented hemorrhagic complications in our investigation is reassuring, aligning with ATLANTIC trial findings and supporting the safety of prehospital ticagrelor administration even in the emergency setting where complete bleeding risk assessment may be limited [27].

The clinical implications of our findings support implementation of prehospital DAPT protocols in STEMI systems of care, recognizing the practical challenges involved in EMS integration [28]. Our protocol achieved median FMC‐to‐DAPT times of only 8 min in the prehospital cohort, demonstrating feasibility when appropriate systems are established. Key implementation elements encompass paramedic training for STEMI recognition, real‐time ECG transmission for physician confirmation, medication availability on ambulances, and clear protocols addressing contraindication screening [29]. The advantages observed in our investigation suggest that even relatively modest extensions of the DAPT‐to‐balloon interval through prehospital initiation translate into meaningful improvements in reperfusion quality and clinical outcomes.

In our multivariable analysis, diabetes mellitus was not independently associated with impaired post‐PCI reperfusion (OR = 0.62, 95% CI: 0.36–1.08, *p* = 0.092). This finding differs from the ATLANTIC trial analysis, in which diabetes mellitus was identified as an independent predictor of suboptimal post‐PCI reperfusion [30]. Several explanations may account for this discrepancy. First, differences in study design and patient characteristics may have influenced the observed associations, as our cohort consisted exclusively of contemporary STEMI patients treated with ticagrelor‐based DAPT, whereas ATLANTIC included a broader randomized population with different baseline thrombotic profiles. Second, improvements in diabetes management and PCI techniques over time may have attenuated the adverse impact of diabetes on reperfusion outcomes. Finally, diabetes was not significantly associated with TIMI 3 flow in our adjusted model, which may be due to insufficient statistical power or differences in baseline glycemic control compared to the ATLANTIC trial, indicating insufficient statistical evidence for an independent association. Therefore, our findings should be interpreted cautiously and require validation in larger prospective cohorts.

Several limitations of this investigation warrant consideration. First, the retrospective observational design introduces potential selection bias, as patients receiving prehospital DAPT may have systematically differed from those receiving in‐hospital therapy in unmeasured characteristics. We addressed this through propensity score matching, which confirmed the robustness of findings, though residual confounding cannot be excluded [31]. Second, group allocation was determined by EMS transport characteristics rather than randomization, potentially introducing confounding by indication. Third, the single‐center design may limit generalizability to other settings with different EMS systems or patient populations. Fourth, TIMI flow assessment, while performed by blinded reviewers, has inherent subjectivity, and we did not perform quantitative coronary flow analyses [32]. Finally, our 30‐day follow‐up period may not capture longer‐term outcomes that could further differentiate treatment strategies [33].

In conclusion, this retrospective cohort investigation demonstrates that prehospital administration of a ticagrelor loading dose in conjunction with aspirin among STEMI patients undergoing primary PCI correlates with substantially elevated rates of optimal coronary reperfusion (TIMI flow grade 3), superior myocardial perfusion indices, and diminished 30‐day MACE compared to in‐hospital DAPT initiation. These advantages were achieved without augmented hemorrhagic risk. Our findings support the implementation of prehospital DAPT protocols in EMS systems and reinforce the principle that earlier antiplatelet therapy translates into improved outcomes in STEMI. Prospective randomized trials with larger time differentials between treatment strategies are warranted to definitively establish the advantages of prehospital ticagrelor administration.

## Author Contributions

We declare that all the listed authors have participated actively in the study and all meet the requirements of the authorship. P.W.L. designed the study and wrote the paper, G.S.C. undertook the data acquisition, YMW managed the literature searches, Q.R.H. undertook the data analysis. All authors reviewed the manuscript.

## Funding

The authors have nothing to report.

## Conflicts of Interest

The authors declare no conflicts of interest.

## Data Availability

The data that support the findings of this study are available from the corresponding author upon reasonable request.
